# Use of Virtual Reality-Based Games to Improve Balance and Gait of Children and Adolescents with Sensorineural Hearing Loss: A Systematic Review and Meta-Analysis

**DOI:** 10.3390/s23146601

**Published:** 2023-07-22

**Authors:** Renato S. Melo, Andrea Lemos, Alexandre Delgado, Maria Cristina Falcão Raposo, Karla Mônica Ferraz, Rosalie Barreto Belian

**Affiliations:** 1 Post-Graduate Program on Child and Adolescent Health, Universidade Federal de Pernambuco (UFPE), Recife 50670-901, PE, Brazil; 2 Laboratory of Informatics in Health, Laboratório de Imunopatologia Keizo Asami (LIKA), Recife 50670-901, PE, Brazil; 3 Department of Physical Therapy, Universidade Federal de Pernambuco (UFPE), Recife 50740-560, PE, Brazil; 4 Laboratory of Pediatric Studies (LEPed), Universidade Federal de Pernambuco (UFPE), Recife 50740-560, PE, Brazil; 5 Instituto de Medicina Integral Professor Fernando Figueira (IMIP), Recife 50070-550, PE, Brazil; 6 Department of Statistics, Universidade Federal de Pernambuco (UFPE), Recife 50740-540, PE, Brazil; 7 Department of Medicine, Universidade Federal de Pernambuco (UFPE), Recife 50670-901, PE, Brazil

**Keywords:** child, deaf, deafness, exercise therapy, exergaming, hearing impairment, motor skills disorders, rehabilitation, vestibular disease, walking

## Abstract

Background: Children and adolescents with sensorineural hearing loss (SNHL) often experience motor skill disturbances, particularly in balance and gait, due to potential vestibular dysfunctions resulting from inner ear damage. Consequently, several studies have proposed the use of virtual reality-based games as a technological resource for therapeutic purposes, aiming to improve the balance and gait of this population. Objective: The objective of this systematic review is to evaluate the quality of evidence derived from randomized or quasi-randomized controlled trials that employed virtual reality-based games to enhance the balance and/or gait of children and adolescents with SNHL. Methods: A comprehensive search was conducted across nine databases, encompassing articles published in any language until 1 July 2023. The following inclusion criteria were applied: randomized or quasi-randomized controlled trials involving volunteers from both groups with a clinical diagnosis of bilateral SNHL, aged 6–19 years, devoid of physical, cognitive, or neurological deficits other than vestibular dysfunction, and utilizing virtual reality-based games as an intervention to improve balance and/or gait outcomes. Results: Initially, a total of 5984 articles were identified through the searches. Following the removal of duplicates and screening of titles and abstracts, eight studies remained for full reading, out of which three trials met the eligibility criteria for this systematic review. The included trials exhibited a very low quality of evidence concerning the balance outcome, and none of the trials evaluated gait. The meta-analysis did not reveal significant differences in balance improvement between the use of traditional balance exercises and virtual reality-based games for adolescents with SNHL (effect size: −0.48; [CI: −1.54 to 0.57]; *p* = 0.37; I^2^ = 0%). Conclusion: Virtual reality-based games show promise as a potential technology to be included among the therapeutic options for rehabilitating the balance of children and adolescents with SNHL. However, given the methodological limitations of the trials and the overall low quality of evidence currently available on this topic, caution should be exercised when interpreting the results of the trials analyzed in this systematic review.

## 1. Introduction

Permanent hearing loss impacts around 62 million individuals under the age of 15 worldwide [1], with approximately 41 million of those residing in developing countries, accounting for two-thirds of the affected population [2]. The prevalence of hearing loss during childhood ranges from 1 to 3 per 1000 live births in developed countries and increases to 6 per 1000 in developing countries [3,4,5]. Sensorineural disorders account for approximately 90% of permanent hearing deficits in children, while conduction disorders and mixed disorders represent 5% each [6,7].

Sensorineural hearing loss (SNHL) refers to a type of hearing impairment that primarily affects the structures within the inner ear. Specifically, SNHL entails damage to the cells of the cochlea, the cochlear nerve, or both [8]. It is worth noting that the cochlea and the vestibule share a continuous membranous labyrinth within the inner ear. Consequently, injuries or traumas that occur prenatally, perinatally, or postnatally can potentially result in damage to either one or both of these organs [9]. As a result, children and adolescents with SNHL are more prone to experiencing peripheral vestibular dysfunctions attributable to inner ear injury.

Peripheral vestibular disorders are commonly observed in children and adolescents with SNHL, with several studies reporting their prevalence ranging from 40% to 89% within their respective sample populations [10,11,12,13,14,15]. These vestibular dysfunctions have the potential to disrupt the balance regulation of individuals with SNHL, leading to alterations in static and/or dynamic balance. It is noteworthy that the vestibular system plays a vital role as the primary sensory organ responsible for the control and regulation of human body balance [16].

Changes in both static and dynamic balance have been documented in children and adolescents with SNHL [16,17,18,19]. Additionally, studies have reported the presence of gait disorders [19,20,21,22,23] and impairments in various other motor skills and competences [24,25,26,27] among this population. These motor disorders can significantly impact the functionality of individuals with SNHL, affecting their engagement in sports, recreational activities, and typical childhood games. Consequently, these children may face challenges in terms of motor performance, making comparisons with their typically hearing peers unfavorable [28]. Such motor difficulties can have implications for their social relationships, increasing the likelihood of emotional disorders and feelings of isolation. It is concerning, as existing evidence indicates that children with SNHL experience higher levels of depressive symptoms, reduced participation in school, sports, and recreational activities, and overall poorer quality of life when compared to their hearing counterparts [29,30,31,32].

Hence, it is crucial to provide timely treatment and rehabilitation for the balance and gait disorders experienced by children with SNHL. Despite extensive research in the field of hearing health, interventions primarily focus on facilitating hearing and communication for children and adolescents with SNHL, often leaving their motor challenges overlooked and neglected [33,34]. Therapeutic motor rehabilitation programs incorporating physiotherapy have been proposed as an effective approach for children with SNHL, yielding satisfactory outcomes [35,36]. However, a common difficulty encountered is that traditional therapeutic exercises may lack appeal for children, leading to reduced adherence and diminished interest in treatment.

In order to enhance children’s engagement in treatment, alternative forms of rehabilitation have been suggested. Some authors have explored the use of sports and recreational activities to improve the balance of children and adolescents with SNHL, and positive effects of these interventions on balance have been observed [37,38]. More recently, virtual environments and the utilization of technological games have been proposed as a rehabilitation approach to enhance the balance of children with hearing loss, demonstrating promising outcomes [39,40]. The authors posit that these technological devices can serve therapeutic purposes for children and adolescents with SNHL as well.

The integration of virtual environments and technological devices into rehabilitation practices has become prevalent in various fields of physiotherapy, particularly in populations with balance and gait impairments [41,42,43,44,45]. In the realm of pediatrics, these devices have been extensively utilized for the assessment and rehabilitation of children and adolescents experiencing balance and gait alterations. They serve as playful and motivating tools for motor and psychological rehabilitation, rendering the rehabilitation environment joyful, enjoyable, and appealing to children [46,47,48,49].

Children with balance and gait impairments, such as those with cerebral palsy, have demonstrated improvement in these motor skills through interventions utilizing virtual reality-based games [50,51]. These findings indicate the effectiveness of such interventions in enhancing the balance and gait of children [52,53,54,55]. Despite the existence of trials employing virtual reality-based games to improve the balance of children and adolescents with SNHL, systematic reviews on this specific topic have not yet been published. Such reviews could offer theoretical and scientific support, justifying the incorporation of these technological devices as additional therapeutic resources for the rehabilitation of balance and gait in children with SNHL. Furthermore, they could provide guidance and promote evidence-based clinical practice in this area, underscoring the significance of conducting this study.

Based on these considerations, the objective of this systematic review was to evaluate the quality of evidence derived from randomized or quasi-randomized controlled trials that employed virtual reality-based games as interventions to enhance the balance and/or gait of children and adolescents with SNHL.

## 2. Materials and Methods

This is a systematic review that was performed according to the guidelines of the Preferred Reporting Items for Systematic Reviews and Meta-Analyses (PRISMA) Statement [56], and its protocol was registered previously on PROSPERO under the number (CRD42018096309) [57].

### 2.1. Identification and Selection of Tests

Searches for articles in this systematic review took place in nine electronic databases: MEDLINE/PubMed, EMBASE, SCOPUS, CENTRAL (Cochrane Central Register of Controlled Trials), LILACS, CINAHL, Web of Science, PEDro, and Google Scholar. The last search was carried out on 1 July 2023, with no restrictions on language or time for publication of the articles. A manual search was also carried out in the list of references of the included trials, to ensure that all relevant trials on the topic were included. The search strategies used in each database are available in Supplementary Materials.

The articles found in each database were independently analyzed by each of the two reviewers (Melo RS and Delgado A), who judged the relevance of the articles by reading the titles and abstracts, in front of a computer, according to the following eligibility criteria: The studies should be randomized or quasi-randomized controlled trials that used virtual reality-based games as an intervention; the volunteers should be children and/or adolescents with clinical diagnosis of bilateral SNHL, ranging in age from 6 to 19 years, without physical problems and cognitive or neurological deficits, except for vestibular dysfunction; and the evaluated outcomes should be balance and/or gait.

In this first analysis, the articles were divided into those eligible or ineligible for this review. Articles with dubious abstracts or with the potential to be included in this systematic review were retained for further analysis by reading the full text of the article. Where the two reviewers disagreed over the inclusion or exclusion of one of the trials for this systematic review, the opinion of a third reviewer (Belian RB) was obtained.

For the articles in which there was a lack of data, the authors of this review sent an email to the corresponding authors of the trials, to obtain the necessary information.

### 2.2. Evaluation of the Characteristics of the Trials

#### Quality of Evidence

The quality of the evidence from the trials was assessed using the GRADE approach [58]. According to this proposal, five factors can interfere with the quality of the evidence of a trial: risk of bias, inconsistency, indirectness, imprecision, and publication bias. For each of these items, the evidence was considered according to the following classification: no (no reduction of points), serious (reduction of 1 point), and very serious (reduction of 2 points), being scored according to the level of severity of risk of bias of the trials.

For the specific GRADE risk of bias item, in order to assess the risk of bias in the trials, we used the Cochrane instrument, which assesses the following items: randomization, allocation secrecy, blinding of volunteers and outcome assessors, losses or incomplete data, selective description of the outcome and others (if applicable). Each item of the risk of bias instrument was assessed in the trials, with the following opinion being attributed: low risk of bias (green), unclear risk of bias (yellow), and high risk of bias (red), according to the risk of bias present in each trial [59].

### 2.3. Participants

The inclusion criteria for the trials encompassed participants who were children and/or adolescents diagnosed with bilateral SNHL, ranging in age from 6 to 19 years. Both control and intervention groups were considered, and individuals with physical impairments, cognitive deficits, and/or associated neurological disorders, except for vestibular dysfunction, were excluded.

### 2.4. Interventions

The intervention group in the selected trials utilized virtual reality-based games of various types, including consoles, platforms, glasses, or virtual helmets. These technological games involve head and body movements and postures that actively engage the children’s vestibular system. They are commonly employed in pediatric physiotherapy to facilitate balance rehabilitation and enhance children’s motor skills. As for the control group, the proposed interventions may have entailed activities of daily living, recreation, leisure, alternative interventions, or even no intervention.

### 2.5. Evaluated Outcomes

The outcomes assessed in this systematic review were: balance (primary outcome) and gait (secondary outcome).

Trials that evaluated the balance outcome by the oscillation speed of the pressure center, in the anteroposterior and mediolateral direction, or by the area of oscillation of the pressure center, by force platform, or computerized dynamic posturography were included.

In addition to these, the trials that assessed balance by the following instruments were also included: pediatric reach test (front or side), test of gross motor of development, pediatric balance scale, one leg standing test, motor proficiency test, Peabody developmental motor scale and movement assessment battery for children (MABC), or any other. All of these clinical tests or scales are widely used in clinical practice and in studies that have assessed balance in children.

For the gait outcome, the trials that evaluated this outcome with any condition related to walking were included, such as gait speed, distance from feet, the width of steps or any other means of locomotion used by the authors. Also included were the trials that evaluated gait with the following instruments: dynamic gait index, timed up and go, accelerometry with the use of video cameras, or that used materials such as paints to mark the footprints of children, in floors or papers, or those that used sand for that.

### 2.6. Data Extraction and Analysis

The trial data included in this review were extracted and recorded on a standardized form prepared by the authors. These data were filed in the RevMan program, version 5.4, by both reviewers independently for later verification of the information and discussion of possible discrepancies. The risk of bias in this systematic review was described qualitatively.

The meta-analysis was performed using the RevMan software. The homogeneity of the trials was observed through the heterogeneity test, considering the studies homogeneous when the *p*-value assumed a value greater than 0.05, and the heterogeneity index (I^2^) being classified as low heterogeneity with values up to 30%. In the statistical analysis, a meta-analysis with a fixed effect was considered; however, when heterogeneity between the tests included in the meta-analysis was observed, the random effect was adopted.

We planned to use the mean difference and to compile the trials that did not report their results in mean and standard deviation for continuous data; we estimated the means and their standard deviation using the method proposed by Hozo et al. [60].

## 3. Results

### 3.1. Flow of Trials through the Review

**Figure 1:**
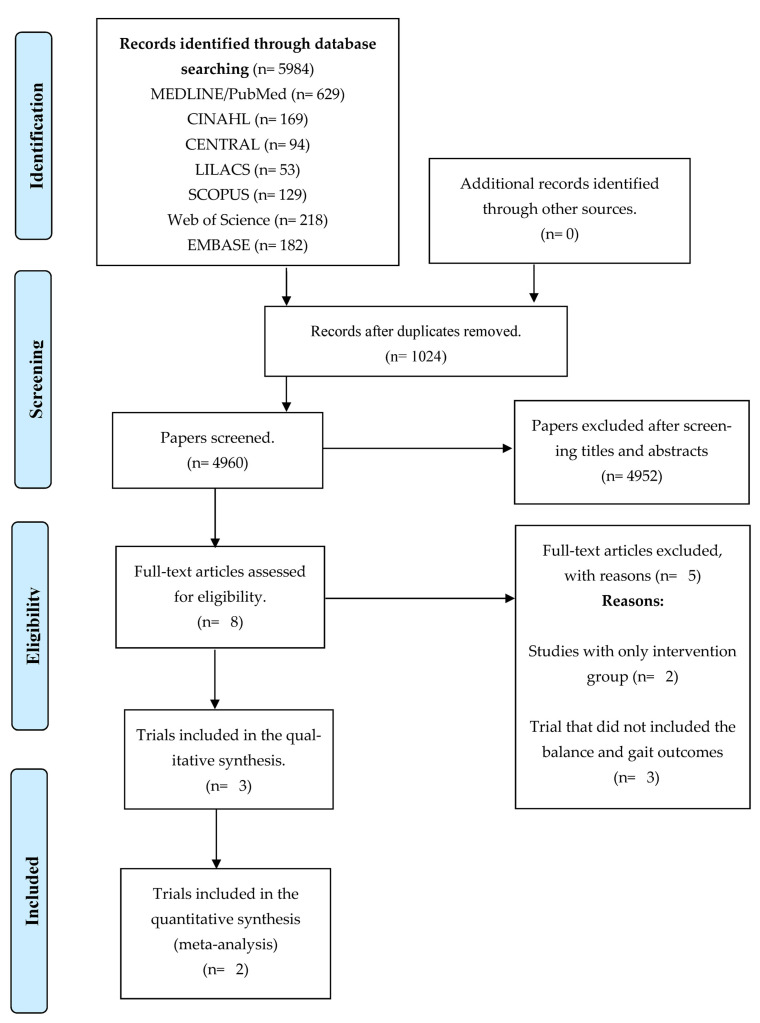
Flowchart of the studies analyzed in this systematic review, according to the Preferred Reporting Items for Systematic Reviews and Meta-Analyses (PRISMA).

A total of 5984 articles were initially identified through the search strategies conducted in the nine databases. After eliminating duplicate articles, 4960 unique articles remained for further analysis. These articles were carefully evaluated based on their titles and abstracts, resulting in the selection of seven articles for full-text reading. Following the comprehensive review, three trials were deemed eligible for inclusion in this systematic review [61,62,63]. Among the selected trials, two were randomized [61,63], while one was quasi-randomized [62]. Figure 1 illustrates the extraction flowchart of the articles included in this review, in accordance with the PRISMA guidelines. Notably, none of the included trials provided information regarding their funding sources.

Among the trials that were excluded from the analysis, two were intervention studies; however, they lacked a control group, thus deviating from the definition of a clinical trial [64,65]. Additionally, three trials did not report relevant outcomes related to balance or gait [66,67,68].

### 3.2. Characteristics of the Included Trials

Within this systematic review, two of the included trials [62,63] involved a comparison between virtual reality-based games and traditional balance exercises conducted during physical education classes. The third intervention [61] employed virtual reality-based games for the intervention group and featured two control groups: one engaged in training with traditional balance exercises, while the third group received no intervention. Table 1 and Table 2 provide detailed information regarding the characteristics of the included trials, including the characterization of the children, methodological aspects, and conclusions.

#### 3.2.1. Risk of Bias

Out of the three trials included in this systematic review, two trials [61,63] reported the use of randomization. However, only Vernadakis et al. [63] provided detailed information on how randomization was performed in their trial, specifically mentioning the generation of random numbers using a computer. On the other hand, Tzanetakos et al. [62] did not mention the utilization of randomization and only stated that the sample was divided into control and intervention groups. This lack of information regarding randomization in Tzanetakos et al.’s trial poses a high risk of bias.

None of the included trials reported the implementation of sample allocation concealment, indicating a high risk of bias for all three trials. Additionally, none of the trials mentioned blinding of the children or outcome assessors, suggesting that the assessors were likely aware of the group assignment of the assessed children (control or intervention). This lack of blinding of outcome evaluators introduces a high risk of bias across all three trials.

It is worth noting that there were no sample losses reported in any of the trials, and none of the trials displayed selective outcome reporting. This ensures a more reliable assessment of the outcomes included in this review.

Another potential bias that was not adequately controlled for in the included trials was the presence of vestibular dysfunction in children. Since changes in balance and gait in children and adolescents with SNHL are frequently attributed to peripheral vestibular disorders resulting from inner ear damage, it is crucial to consider and account for this factor in the assessment of outcomes. Failure to control for vestibular dysfunction could potentially influence the results and interpretation of the findings.

**Figure 2:**
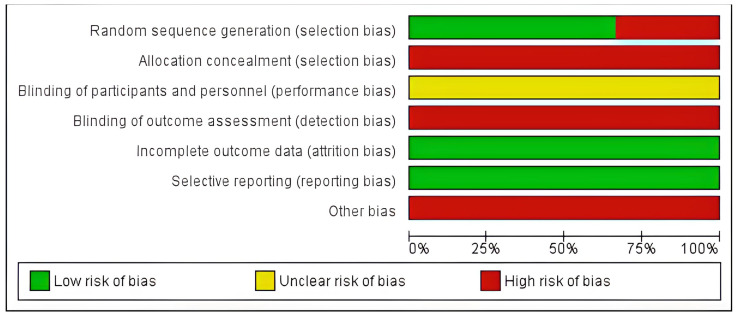
Risk of bias summary of the included trials assessed using the Cochrane risk-of-bias tool.

**Figure 3:**
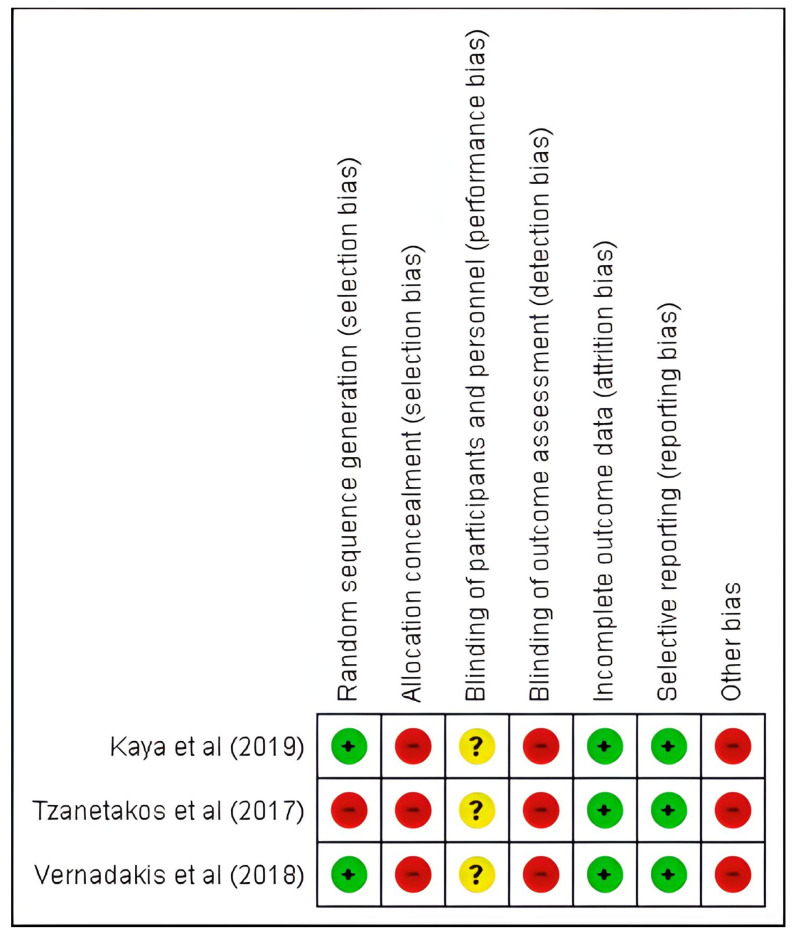
Risk of bias of each included trial assessed using the Cochrane risk-of-bias tool [61,62,63].

Moreover, it is worth noting that children and adolescents with SNHL who also have associated vestibular dysfunction tend to exhibit the most compromised balance performance. Failing to control for the presence of vestibular dysfunction in the sample could potentially lead to an underestimation of the intervention effects, introducing a high risk of bias in the trials. This observation is supported by the findings presented in Figure 2 and Figure 3, as well as Table 3, which provide a critical analysis of the overall and isolated risk of trial bias and the GRADE evidence quality table, respectively.

#### 3.2.2. Participants

The three trials included in this review involved a total of 66 volunteers and observed the effectiveness of using virtual reality-based games to improve the balance of adolescents with SNHL.

#### 3.2.3. Intervention

All three trials included in this systematic review utilized virtual reality-based games as interventions, specifically using the Nintendo Wii^®^ console with the Wii Balance Board. Kaya et al. [61] divided their sample into three groups. Intervention Group 1 received balance training involving exercises with head and trunk movements, as well as the single leg support test. Intervention Group 2 used virtual reality-based games, specifically Bubble Balance and Ski Slalom. Both interventions were conducted three times per week, with each session lasting 30 min, over a duration of 8 weeks. The third group did not undergo any balance training. The authors observed improvements in static and dynamic balance among the participants in Groups 1 and 2 following the interventions.

The other two trials, Tzanetakos et al. [62] and Vernadakis et al. [63], employed similar interventions. In both trials, sessions were conducted twice a week, lasting 15 min each, over a period of 5 weeks, for both the control and intervention groups.

In both trials by Tzanetakos et al. [62] and Vernadakis et al. [63], the control group participated in exercises that involved walking on tiptoes, walking along a straight line on the floor, and balancing on one foot on the ground and on inflatable discs. On the other hand, the intervention group utilized various Nintendo Wii Fit Plus games with the Wii Balance Board, including Bubble Balance, Lotus Focus, Penguin Slide, Ski Jump, Ski Slalom, Snowboard Slalom, Soccer Heading, Table Tilt, Tightrope Walk, and Yoga. Both trials reported improvements in balance among adolescents in both the control and intervention groups following the intervention period.

#### 3.2.4. Outcome Measures

Kaya et al. [61] employed a force platform to assess the balance of their sample. Similarly, Tzanetakos et al. [62] and Vernadakis et al. [63] utilized the Flamingo Balance test as the instrument to evaluate balance in their respective samples. The use of the same assessment tool, the Flamingo Balance test, in Tzanetakos et al. [62] and Vernadakis et al. [63] trials enabled the possibility of conducting a meta-analysis to analyze and combine the results of these two trials. However, no trials were found in the literature that included the outcome of gait.

### 3.3. Meta-Analysis

**Figure 4:**
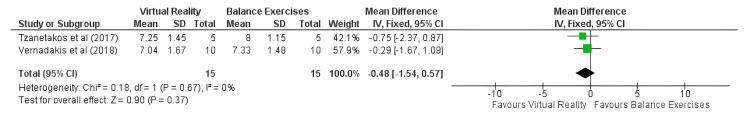
Forest plot of the comparison between the use of virtual reality-based games versus traditional balance exercises to improve the balance of adolescents with SNHL, represented by the number of falls or exits from the Flamingo position, measured by the Flamingo Balance test [62,63].

The meta-analysis conducted in this study did not show any significant differences between using traditional balance exercises and virtual reality-based games in improving the static balance of adolescents with SNHL. The effect size estimate was −0.48 with a confidence interval of −1.54 to 0.57 (*p* = 0.37), and there was no evidence of heterogeneity (I^2^ = 0%). It is important to note that the results of the trials included in the meta-analysis were inversely proportional, meaning that higher values indicated poorer balance performance. The Flamingo Balance test, used in the trials, assessed the number of falls or deviations from the Flamingo position, with a higher number indicating poorer performance on the test (Figure 4).

## 4. Discussion

This is the first systematic review to evaluate the quality of evidence from trials that used virtual reality-based games to improve the balance and gait of children and/or adolescents with SNHL.

Three trials on the topic were analyzed and, although the trials observed that the balance of adolescents with SNHL improved after the interventions, the quality of this evidence is very low, due to the methodological limitations present in the trials.

The main methodological limitations and bias observed in the evaluated trials were related to three categories: sample selection, methodological bias, and bias related to children’s characteristics. So, we decided to score them and discuss them, in isolation, below.

### 4.1. Biases Related to Sample Selection

In the trial conducted by Tzanetakos et al. [62], the process of randomization was not mentioned; randomization is a critical step in clinical trials to ensure comparability between the intervention and control groups, thus minimizing selection bias. Furthermore, none of the trials included in this systematic review addressed the issue of sample allocation secrecy, which is an important methodological aspect to prevent researchers from having prior knowledge of the group allocation. The lack of control in these two crucial stages of a clinical trial, particularly the absence of allocation secrecy, indicates the need for better implementation of these measures in future trials on this topic. It should be noted that trials without adequate allocation secrecy can lead to an overestimation of the intervention’s value by up to 30% [69]. Therefore, it is essential for future studies to prioritize and improve the control of these selection biases.

An additional bias identified in the trial conducted by Kaya et al. [61] pertains to the sample characteristics. The volunteers in this study were active sports practitioners, which may have influenced the outcomes observed. It has been demonstrated in previous research that engaging in sports activities can improve the balance of children and adolescents with SNHL [70,71,72]. Therefore, including individuals who were concurrently involved in sports activities raises the question of whether the observed improvement in balance in the intervention group of Kaya et al.’s trial [61] was attributed to the use of virtual reality-based games or the practice of sports activities that the sample engaged in parallel to the trial.

Although it is not explicitly stated whether the participants in Kaya et al.’s trial [61] continued their sports activities during the study, the authors did not report any intervention to interrupt such activities. Ideally, the trial should have included a sample consisting of untrained individuals who had not previously undergone specific balance training. This would allow for better comparability with other trials that used samples composed of untrained individuals. Failure to consider this aspect may hinder the grouping of results from Kaya et al.’s trial with those from other studies and complicate future meta-analyses, thereby impeding a comprehensive understanding of the true effects of interventions utilizing virtual reality-based games on the balance of children and adolescents with SNHL.

### 4.2. Biases Related to the Methodological Aspects of the Trials

Another important bias identified in the trials reviewed was the lack of blinding of the outcome assessors. This methodological aspect is crucial to ensure the reliability of the findings and minimize biases such as conduction bias and detection bias. Without blinding, there is a risk that prior knowledge of the sample allocation may influence the response to treatment or the evaluation of outcomes. Trials that lack double-blinding have been shown to overestimate the effect of the intervention by approximately 17% [69]. Therefore, it is essential to enforce stricter control and ensure blinding of outcome assessors in future trials on this topic to enhance the quality of evidence and the credibility of the findings.

Additionally, a bias was observed in the trial conducted by Vernadakis et al. [63]. In addition to the intervention period, the intervention group had an extra 120 min introductory session on how to use the virtual reality-based games. This additional session resulted in a longer treatment time for the intervention group compared to the control group. Such disparity in treatment duration may have influenced the trial results. When comparing two treatments, it is essential to ensure that the interventions are similar in terms of session duration, frequency per week, and total intervention period to allow for a fair comparison and determine whether the interventions are equivalent or if one is superior in improving the targeted outcomes.

Therefore, offering the intervention group more time for using virtual reality-based games in the trial by Vernadakis et al. [63] introduced an inconsistency and may have introduced bias in the study design. Future trials should aim for more balanced and comparable intervention protocols to enhance the validity of their findings.

### 4.3. Bias Related to Sample Characteristics

#### Assessment of the Function of the Vestibular System

An important bias observed in the analyzed trials was the lack of control over the presence of vestibular dysfunction in children with SNHL. It is well-established that children and adolescents with SNHL often experience balance disorders associated with vestibular dysfunctions, which are frequently observed due to the inner ear injury.

The presence of vestibular dysfunction can significantly impact balance performance in children with SNHL. Studies have consistently shown that children with SNHL and associated vestibular dysfunction exhibit poorer balance compared to those with normal hearing [73,74,75,76,77,78] and even those with SNHL but without vestibular dysfunction [74,75]. Therefore, when conducting a clinical trial aiming to improve the balance of children with SNHL, it is essential to consider this discrepancy in balance performance.

Children without vestibular dysfunction may respond more quickly to the intervention and demonstrate satisfactory results, while children with vestibular dysfunction may exhibit slower progress due to their greater balance impairment. Thus, it is crucial to acknowledge that children with SNHL with and without vestibular dysfunctions may require different periods of time to achieve balance improvement. Future trials on this topic should carefully consider the presence or absence of vestibular dysfunction in the sample, as it can significantly influence the trial results.

By controlling for the presence of vestibular dysfunction, researchers can gain a better understanding of the effects of interventions on balance outcomes and tailor interventions accordingly to address the specific needs of children with SNHL and associated vestibular dysfunctions.

The possible influence of vestibular dysfunction on the trial outcomes, particularly in the trial by Tzanetakos et al. [62], suggests that the intervention group may have included children with vestibular dysfunctions who required a longer intervention time than what was provided in the trial (15 min). However, since the authors did not evaluate the vestibular function of the sample, this remains a hypothesis that can be confirmed or refuted by future trials.

Furthermore, it is noteworthy that the samples in the trials by Tzanetakos et al. [62] and Vernadakis et al. [63] consisted of adolescents with higher degrees of hearing loss. Numerous studies have established an association between the extent of hearing loss and the presence of vestibular dysfunction in children and adolescents with SNHL [79,80,81,82]. This increases the likelihood that these trials included a significant number of children with vestibular dysfunction, which was not controlled for. Hence, it is crucial to assess the vestibular function of children in future trials on the topic.

The presence or absence of vestibular dysfunction in children with SNHL can indeed influence the trial results. The inclusion of many children without vestibular dysfunction in the sample can overestimate the intervention effect, while the inclusion of a substantial number of children with vestibular disorders can underestimate the intervention effect. Consequently, future trials should consider this aspect during sample allocation at the time of randomization. One suggestion is to employ a block randomization strategy that accounts for children with and without vestibular disorders, thereby ensuring homogeneity in the distribution of children based on their vestibular function in both the control and intervention groups. By doing so, authors will be able to examine the effects of interventions in more homogeneous samples with respect to the vestibular function of children, leading to a better-controlled bias.

By addressing the influence of vestibular dysfunction through proper sample allocation and evaluation, researchers can enhance the quality of evidence and provide more accurate insights into the effects of interventions using virtual reality-based games on the balance of children and adolescents with SNHL.

Controlling for vestibular dysfunction is indeed crucial, as it can impact the duration and effectiveness of interventions. This information holds value for both future trials and clinical practice. Trials with longer intervention durations may achieve positive effects for children with SNHL, regardless of whether they have vestibular disorders. On the other hand, trials with shorter intervention durations may primarily benefit children without vestibular dysfunctions, who have less severe balance impairments. Therefore, it is important to assess the vestibular function of children and adolescents with SNHL in future trials on the topic.

The hypotheses mentioned above can only be confirmed or rejected through future trials that control for vestibular dysfunction and include children with and without vestibular disorders. Unfortunately, none of the analyzed trials evaluated the vestibular function of their samples. Thus, it is crucial to include an assessment of vestibular function in future trials to gain insights into the effects of interventions on children with and without vestibular dysfunctions.

Failure to control for vestibular dysfunction in the sample can confound the trial results. Without controlling for vestibular dysfunction, it is not possible to generalize the effects of interventions to children with vestibular disorders, who experience greater balance impairments. Therefore, it is essential to assess and control for vestibular dysfunction in the sample.

In addition to evaluating the vestibular function of the sample, future trials should report their results separately, dividing the sample into groups based on the presence or absence of vestibular disorders. This would provide a better understanding of the effects of interventions on children with and without vestibular disorders, helping guide clinical practice.

When assessing the vestibular system, De Kegel et al. [73] suggest that the asymmetry of the vestibular evoked myogenic potential (VEMP) could serve as a good predictor of balance disorders in children with SNHL. Additionally, the video head impulse test (vHIT) has been found to be a convenient, quick, and comfortable method for assessing the vestibular function of children with SNHL as young as 3 years old [83]. Hence, VEMP and vHIT are potential methods that future trials can consider for evaluating the vestibular system of children in their samples.

### 4.4. Other Limitations of the Trials

The systematic review identified several limitations in the analyzed trials. One important limitation was the small sample sizes, and it was unclear whether sample size calculations were performed. By conducting sample size calculations, researchers can ensure that an adequate number of participants are included in the study to detect meaningful effects and improve the generalizability of the findings.

Furthermore, the trials did not mention whether the instruments used to assess the balance of the adolescents were validated. Using validated instruments for outcome assessment is crucial, as they provide more accurate and reliable results. Validated instruments also contribute to standardizing trial results, enabling future meta-analyses to guide clinical practice and decision-making on the topic.

Additionally, none of the analyzed trials reported the presence or absence of adverse effects associated with the interventions. Assessing and reporting adverse effects is essential for a comprehensive understanding of the intervention’s safety profile. This information is valuable for rehabilitation professionals when making informed decisions about the benefits and risks of using virtual reality-based games in the treatment of children and adolescents with SNHL.

To address these limitations, future trials on the topic should consider conducting sample size calculations to ensure adequate statistical power. It is also crucial to use validated instruments for outcome assessment to ensure the accuracy and reliability of the results. Furthermore, researchers should carefully monitor and report any potential adverse effects associated with the interventions. These improvements will contribute to a more robust and informative body of evidence in the field of using virtual reality-based games for improving balance in children and adolescents with SNHL.

### 4.5. Considerations for Future Trials on the Topic

The review identified that the trials analyzed in the review used the Nintendo Wii^®^ console with the Wii Balance Board for the interventions involving virtual reality-based games. However, the effects of these interventions were not as significant for children with SNHL compared to children with cerebral palsy [84,85,86], where previous studies have shown improvements in balance and gait.

One possible explanation for this difference in effectiveness could be the greater motor impairment observed in children with cerebral palsy compared to children with SNHL. Children with cerebral palsy often struggle with maintaining an upright posture and mobility, and the motor tasks required by virtual games on the Wii Balance Board can serve as a form of vestibular rehabilitation and balance training for these children.

In contrast, balance and gait impairments in children with SNHL are generally more subtle, and the balancing and movement tasks performed on the Wii Balance Board may not provide as effective stimulation to the vestibular system and tonic-postural reflexes in children with milder motor deficits. It is possible that using a different type of console that allows for greater mobility and wider displacements of the center of gravity, such as the Xbox 360^®^, could yield more significant results and greater effectiveness in improving balance in children with SNHL.

Therefore, it is important for future trials to consider the level of motor deficits in children and the type of stimulus provided by the consoles used in the interventions. The Nintendo Wii^®^ with the Wii Balance Board may be more effective for children with more severe motor deficits, where smaller displacements of the center of gravity can have positive effects on balance. On the other hand, for children with milder motor deficits, such as those with SNHL, using consoles like the Xbox 360^®^ that offer more complex motor tasks and greater displacement of the center of gravity may be a more suitable option to improve balance.

Indeed, the hypotheses presented above regarding the effectiveness of different gaming consoles for balance rehabilitation in children with SNHL are speculative and require further investigation through future trials. While no existing literature has demonstrated the superiority of one console over another for this specific population, the choice of console in previous trials seems to align with the suggested hypotheses.

Trials involving children with more significant motor deficits, such as those with cerebral palsy, have commonly utilized the Nintendo Wii with the Wii Balance Board [87,88]. On the other hand, trials with children with milder motor deficits, like those with SNHL, have employed consoles such as the Xbox 360^®^ [89,90]. However, it is essential to note that these observations are based on existing trials and may not necessarily indicate a definitive correlation between console choice and effectiveness.

In future trials, alternative consoles could be explored, including those integrated with virtual glasses and helmets. Such devices could offer opportunities for balance and gait rehabilitation by encouraging head and eye movements, aligning with key exercises for vestibular rehabilitation in children [91]. Nonetheless, when utilizing virtual glasses and helmets, researchers should consider potential variations in head movement range and postural alignment in children and adolescents with SNHL [92,93,94].

Further research is needed to investigate the comparative effectiveness of different gaming consoles and technological devices in balance rehabilitation for children with SNHL. By exploring various options and considering the specific motor deficits and needs of the target population, future trials can provide valuable insights into optimizing interventions and enhancing the outcomes of balance rehabilitation for children with SNHL.

Future trials on the topic should carefully consider the use of hearing devices, such as hearing aids and cochlear implants, by children with SNHL. It is crucial to control for the presence of these devices, as they can potentially influence the results of balance assessments.

Among the analyzed trials, only Tzanetakos et al. [62] explicitly stated that children with cochlear implants were excluded from their sample, while the other trials did not provide this information. Additionally, none of the trials reported whether the children in their sample were using hearing aids. This information is highly relevant, as it can significantly impact the outcomes of the trials.

Existing evidence suggests that the use of hearing aids and cochlear implants improves balance in adults, and older adults [95,96,97,98,99,100,101], and children with cochlear implants [102,103,104,105,106,107]. These improvements may be attributed to the auditory input provided by these devices, which can serve as reference points and enhance spatial awareness and balance [108,109,110,111,112]. Therefore, including children who use cochlear implants or hearing aids in trials may introduce bias, as the presence of these devices can potentially overestimate the intervention’s effect.

Children who utilize cochlear implants or hearing aids may exhibit enhanced spatial-temporal orientation and balance due to the auditory cues provided by these devices [113,114,115,116]. In addition, these devices have shown satisfactory results on hearing and communication outcomes of children with SNHL [117,118,119,120,121], which could influence the trial results. However, cochlear implants have also been associated with vestibular disorders and balance changes in children with SNHL [122,123,124,125,126,127]. This further underscores the importance of controlling for the use of cochlear implants in future trials, as their presence can introduce additional biases.

To mitigate these biases, future trials should consider including children with SNHL with and without cochlear implants separately and report their results independently. This approach would allow for a more accurate assessment of the intervention’s effectiveness for each group, reducing the potential influence of hearing devices on the outcomes.

In summary, the use of hearing aids and cochlear implants by children with SNHL can impact balance and may introduce biases in trials. To minimize these biases, future trials should consider stratifying the sample based on the presence or absence of cochlear implants and hearing aids and report results separately for each group. This will provide valuable insights into the effectiveness of interventions for children with and without hearing devices, guiding clinical practice and decision-making in balance rehabilitation for children with SNHL.

There is a need for future trials that specifically focus on improving the balance, motor skills, functional abilities, quality of life, and well-being of children with cochlear implants. Despite the existing evidence suggesting that children with cochlear implants may experience vestibular disorders and balance changes, trials targeting balance improvement in this population are lacking. It is crucial to develop interventions that address the unique needs of children with cochlear implants and aim to enhance their balance and overall motor function.

Despite the biases discussed earlier, the analyzed trials in this review have several positive aspects that should be retained and replicated in future studies. One important aspect is the detailed description of the exercises performed by the children, which allows for replicability in subsequent trials and enables rehabilitation professionals to incorporate these exercises into their clinical practice. By providing clear guidelines and descriptions, future trials can ensure consistency and comparability in intervention protocols.

Another positive aspect observed in the study conducted by Tzanetakos et al. [62] was the inclusion of parents and members of the school community in the evaluation process for virtual reality-based games. Involving parents in therapy and seeking their input is essential for a child-centered approach and can provide valuable insights for future therapeutic decision-making. Maintaining this collaborative approach in future trials will further enhance the effectiveness and acceptance of interventions.

This systematic review highlighted that virtual reality-based games can positively impact the balance of children with SNHL when used in interventions with a session duration of 30 min or more, three times a week, for 8 weeks [61]. This information can serve as a guideline for future trials, supporting the recommendation of incorporating virtual reality-based games into balance rehabilitation programs for children with SNHL.

It is important to acknowledge that this systematic review did not include searches in thesis and dissertation databases, which is a limitation of the study. Future research should consider expanding the search scope to include these additional sources of relevant information.

As evident from the literature, children with SNHL often experience balance and gait disorders that stem from delays in reaching developmental milestones [128,129,130,131,132,133]. Without appropriate interventions or treatments, these challenges can persist from childhood into adolescence [134,135,136,137,138,139]. This underscores the critical importance of early assessment and physical therapy interventions for children with SNHL, ideally starting from infancy or as soon as the diagnosis of SNHL and/or vestibular dysfunction is made. Implementing hearing and vestibular screening programs [140] and promptly referring affected children for evaluation and physiotherapy treatment are vital in addressing their needs effectively.

## 5. Conclusions and Implications for Future Trials on the Topic

The use of virtual reality-based games shows promise as a therapeutic option for balance rehabilitation in children and adolescents with SNHL. However, due to the methodological limitations and low quality of the current evidence, caution should be exercised when interpreting the results of the trials analyzed in this systematic review.

The question of whether the use of virtual reality-based games improves balance and gait in children and adolescents with SNHL remains inconclusive due to the low quality of the evidence. Furthermore, the lack of reporting on adverse effects of interventions makes it challenging to assess the balance between benefits and potential risks.

To establish the effectiveness of virtual reality-based games for improving balance and gait in this population, it is recommended that new trials with stronger methodological rigor be conducted. These trials should address sample selection bias, employ blinding techniques for outcome evaluators, assess the function of the children’s vestibular system, and utilize validated instruments to assess outcomes.

Additionally, future trials should investigate not only balance but also other functional and clinical outcomes such as gait, functionality, quality of life, fall frequency, vertical jump, activities of daily living, sports performance, recreational activities, and social interaction. These aspects have been overlooked in the existing literature on the topic.

Moreover, it would be valuable for future trials to explore the optimal duration of sessions, frequency of interventions, types of games, and the effectiveness of different consoles for balance and gait rehabilitation in children with SNHL, with and without vestibular dysfunctions. Long-term effects and the satisfaction of parents and children with the interventions should also be assessed, including improvements in specific domains of daily life skills. Understanding the preferences and perspectives of both parents and children will facilitate a child-centered approach to therapy and inform future decision-making.

By addressing these research gaps, future trials can provide robust scientific evidence to guide clinical practice and ensure that motor rehabilitation utilizing virtual reality-based games for children and adolescents with SNHL is based on high-quality evidence.

## Figures and Tables

**Table 1:** Summary of the included trials.

Author	Country	Motor Skill	Design	Characteristics of the Volunteers	Number of Volunteers		Characteristics of the Interventions	
					CG	IG	CG	IG
Kaya et al. [61]	Turkey	Balance	RCT	Adolescents with SNHL, male sex, and age range between 18–22 years old.	24	12	CG1: 30 min sessions, three times/week of balance training, for 8 weeks. CG2: Control group was not given any balance training.	IG: 30 min sessions, three times/week of practice with active videogames, for 8 weeks.
Tzanetakos et al. [62]	Greece	Balance	Quasi-randomized trial	Adolescents with profound SNHL (hearing loss degrees > 70 dB), both sexes and age range between 17–19 years old.	05	05	15 min sessions, two times/weeks of balance training, for 5 weeks.	15 min sessions, two times/week of practice with active videogames, for 5 weeks.
Vernadakis et al. [63]	Greece	Balance	RCT	Adolescents with profound SNHL (hearing loss degrees > 70 dB), both sexes and age range between 17–19 years old.	10	10	15 min sessions, two times/weeks of balance training, for 8 weeks.	15 min sessions, two times/week of practice with active videogames, for 8 weeks.

RCT: Randomized controlled trial; SNHL: Sensorineural hearing loss; IG: Intervention group; CG: Control group; dB: Decibels.

**Table 2:** Methodological aspects and conclusions of the trials that used virtual reality-based games to improve the balance of adolescents with SNHL.

Author	Motor Skill	Outcome Measures	Instruments Used for the Assessment	Control Group		Intervention Group		Conclusions
				Pre	Post	Pre	Post	
Kaya et al. [61]	Balance	Static Balance	Force Platform	CG1: 0.68 ± 0.18 ^a^ CG2: 0.82 ± 0.10	CG1: 0.60 ± 0.13 ^a^ CG2: 0.81 ± 0.07	IG: 0.79 ± 0.17 ^b^	IG: 0.67 ± 0.11 ^b^	Virtual reality-based games were as effective as traditional balance exercises to improve balance of adolescents with SNHL. However, the use of virtual reality-based games is more effective than not exercising to improve the balance of adolescents with SNHL.
		Dynamic Balance		CG1: 1.65 ± 0.26 ^a^ CG2: 1.87 ± 0.28	CG1: 1.46 ± 0.22 ^a^ CG2: 1.78 ± 0.23	IG: 1.78 ± 0.36 ^b^	IG: 1.48 ± 0.17 ^b^	
Tzanetakos et al. [62]	Balance	Static Balance	Flamingo Balance Test	9.00 ± 0.58	8.00 ± 1.15	7.75 ± 1.51	7.25 ± 1.45	Balance exergames constitute a feasible, well-accepted and motivational balance training mode for adolescents with deafness, the effectiveness of which should be further researched.
Vernadakis et al. [63]	Balance	Static Balance	Flamingo Balance Test	9.09 ± 0.74	7.04 ± 1.67	9.21 ± 0.71	7.33 ± 1.48	Findings support the effectiveness of using the Nintendo Wii gaming console as an intervention for adolescent students with deafness, and specifically, its effects on physical function related to balance competence.

^a^: Data regarding the intervention using traditional balance exercises; ^b^: Data regarding the intervention with virtual reality-based games in the trial; IG: Intervention group; CG: Control group.

**Table 3:** Quality of the evidence of the trials that used virtual reality-based games to improve the balance of children and adolescents with SNHL.

Quality Assessment							№ of Patients			Effect	Quality of the Evidence	Importance
№ of Studies	Study Design	Risk of Bias	Inconsistency	Indirectness	Imprecision	Other Considerations	Virtual Reality	Balance Exercises	Relative (95% CI)	Absolute (95% CI)		
Balance: (follow up: mean 8 weeks; assessed with Force Platform)												
01 [61]	RCT	very serious ^b,c,d,e^	serious ^f^	not serious	not serious	none	12	12	-	-	⨁◯◯◯ VERY LOW	CRITICAL
Balance: (follow up: mean 7 weeks; assessed with Flamingo Balance Test)												
02 [62,63]	QRT	very serious ^a,b,c,d,e^	very serious ^g^	not serious	not serious	none	15	15	-	−0.48 [−1.54 to 0.57]	⨁◯◯◯ VERY LOW	CRITICAL

RCT: Randomized controlled trial; QRT: Quasi-randomized trial; ^a^: There was no random sequence generation; ^b^: No allocation secrecy; ^c^: There was no blinding of the children; ^d^: There was no blinding of the evaluator of the outcomes; ^e^: There was no evaluation of children’s vestibular function; ^f^: Data from a single trial; ^g^: Longer intervention time for the intervention group.

## Data Availability

All data from this study are available in this article.

## Supplementary Materials

The search strategy is provided as supplementary material at: https://www.mdpi.com/article/10.3390/s23146601/s1.
