# Supplementary material for: Use of Virtual Reality-Based Games to Improve Balance and Gait of Children and Adolescents with Sensorineural Hearing Loss: A Systematic Review and Meta-Analysis
Source: Sensors (Basel). 2023 Jul 22;23(14):6601. doi: 10.3390/s23146601 (PMC10385194; doi:10.3390/s23146601)
Supplement: Supplementary file 1 [file sensors-23-06601-s001.zip › sensors-2473331-supplementary.pdf]

Use of Virtual Reality-Based Games to Improve Balance and Gait of Children and Adolescents with Sensorineural Hearing Loss: A Systematic Review and Meta-Analysis

Search Strategies

MEDLINE/PubMed

(pediatr*[tiab] OR child[mh] OR children[tiab] OR Adolescent[mh] OR Adolescent*[tiab] OR teen*[tiab] OR teenager[tiab] OR child*[tiab] OR infant[tiab]) AND ("hearing impairment"[mh] OR "hearing impairment"[tiab] OR "impaired hearing"[tiab] OR "auditory defect"[tiab] OR "hearing damage"[tiab] OR "hearing defect"[tiab] OR "bilateral deafness"[tiab] OR "deaf"[tiab] OR "deafness"[tiab] OR "hearing loss"[tiab] OR "perception deafness"[tiab] OR "deafness sensorineural"[tiab] OR "hearing loss sensorineural"[tiab] OR "neurosensory deafness"[tiab] OR "neurosensory hearing impairment"[tiab] OR "neurosensory hearing loss"[tiab] OR "sensori-neural deafness"[tiab] OR "sensori-neural hearing impairment"[tiab] OR "sensori-neural hearing loss"[tiab] OR "sensorineural deafness"[tiab] OR "sensorineural hearing impairment"[tiab] OR "sensorineural hearing loss"[tiab] OR "vestibular disorder"[tiab] OR "disorder vestibular"[tiab] OR "vestibular diseases"[mh] OR "vestibular disease"[tiab] OR "vestibular disorder"[tiab] OR "vestibular disturbance"[tiab] OR "vestibular dysfunction"[tiab] OR "vestibular impairment"[tiab] OR "vestibulopathy"[tiab]) AND ("Track and Field"[mh] OR "Track and Field"[tiab] OR "Racquet Sports"[mh] OR "Racquet Sports"[tiab] OR Basketball[mh] OR "Basket ball"[tiab] OR Basketball[tiab] OR boxing[tiab] OR boxing[mh] OR "aquatic sports"[tiab] OR "aquatic sport"[tiab] OR "water sports"[tiab] OR watersport*[tiab] OR Bicycling[mh] OR Bicycling[tiab] OR cycling[tiab] OR "fencing (sport)"[tiab] OR "fencing sport"[tiab] OR soccer[mh] OR soccer[tiab] OR "artistic gymnastics"[tiab] OR "rhythmic gymnastics"[tiab] OR Golf[mh] OR Golf[tiab] OR handball[tiab] OR equestrian[tiab] OR Hockey[mh] OR Hockey[tiab] OR icehockey[tiab] OR "Martial Arts"[tiab] OR "Martial Arts"[mh] OR "martial art"[tiab] OR "martial sport"[tiab] OR "Weight Lifting"[mh] OR "Weight Lifting"[tiab] OR Wrestling[mh] OR Wrestling[tiab] OR Swimming[mh] OR Swimming[tiab] OR "Water Sports"[mh] OR "Water Sports"[tiab] OR "Water Sport"[tiab] OR pentatl*[tiab] OR football[mh] OR football[tiab] OR rugby[tiab] OR taekwondo[tiab] OR "taekwon-do"[tiab] OR "tae kwon do"[tiab] OR "pistol sport"[tiab] OR Tennis[mh] OR Tennis[tiab] OR "ping-pong"[tiab] OR triatlon[tiab] OR triathlon[tiab] OR Volleyball[mh] OR Volleyball[tiab] OR bobsleigh[tiab] OR bobsled[tiab] OR bobsledge[tiab] OR biathlon[tiab] OR curling[tiab] OR "alpine ski"[tiab] OR "cross-country"[tiab] OR ski[tiab] OR Hockey[tiab] OR luge[tiab] OR "ice skating"[tiab] OR "speed skating"[tiab] OR "Ski jumping"[tiab] OR skeleton[tiab] OR Skiing[mh] OR Skiing[tiab] OR Snowboarding[tiab] OR Dancing[mh] OR ballet[tiab] OR dancing[tiab] OR Slacklining[tiab] OR Slackline[tiab] OR Exergames[tiab] OR Virtual Reality[tiab] OR Virtual Reality Exposure Therapy[tiab] OR capoeira[tiab] OR "rope skipping"[tiab] OR bike[tiab] OR bicycle[tiab] OR "Roller skating"[tiab] OR rollerskating[tiab] OR skateboard[tiab] OR skateboarding[tiab] OR skating[mh] OR skating[tiab] OR surf[tiab] OR sport[majr])

EMBASE

('adolescent'/exp OR 'adolescent' OR 'teenager' OR 'child'/exp OR 'child' OR 'children' OR 'infant'/exp OR 'infant') AND ('hearing impairment'/exp OR 'auditory defect' OR 'bilateral deafness' OR 'deaf' OR 'deafness' OR 'deafness, bilateral' OR 'hearing damage' OR 'hearing defect' OR 'hearing impairment' OR 'hearing loss' OR 'hearing loss, bilateral' OR 'hearing loss, central' OR 'hearing loss, high frequency' OR 'impaired hearing' OR 'perception deafness'/exp OR 'deafness, sensorineural' OR 'neurosensory deafness' OR 'neurosensory hearing impairment' OR 'neurosensory hearing loss' OR 'sensori-neural hearing impairment' OR 'sensori-neural hearing loss' OR 'sensorineural deafness' OR 'sensorineural hearing impairment' OR 'vestibular disorder'/exp OR 'disorder, vestibular' OR 'vestibular diseases' OR 'vestibular disease' OR 'vestibular disturbance' OR 'vestibular dysfunction' OR 'vestibular impairment' OR 'vestibulopathy') AND

('track and field'/exp OR 'track and field' OR 'racquet sport'/exp OR 'racket sport' OR 'racket sports' OR 'racquet sport' OR 'racquet sports' OR 'basketball'/exp OR 'basket ball' OR 'basketball' OR 'boxing'/exp OR 'boxing' OR 'aquatic sport'/exp OR 'aquatic sport' OR 'sports, aquatic' OR 'water sport' OR 'water sports' OR 'watersport' OR 'cycling'/exp OR 'bicycling' OR 'cycling' OR 'fencing (sport)'/exp OR 'fencing (sport)' OR 'soccer'/exp OR 'soccer' OR 'socker' OR 'artistic gymnastic' OR 'rhythmic gymnastic' OR 'golf'/exp OR 'golf' OR 'golf ball' OR 'handball'/exp OR 'equestrian'/exp OR 'martial art'/exp OR 'martial art' OR 'martial arts' OR 'martial sport' OR 'weight lifting'/exp OR 'lifting, weight' OR 'weight lifting' OR 'wrestling'/exp OR 'wrestling' OR 'swimming'/exp OR 'swimming' OR 'swimming test' OR 'football'/exp OR 'football' OR 'rugby'/exp OR 'rugby' OR 'taekwondo'/exp OR 'tae kwon do' OR 'taekwondo' OR 'pistol sport' OR 'tennis'/exp OR 'tennis' OR 'ping pong' OR 'triathlon'/exp OR 'triathlon' OR 'volleyball'/exp OR 'volleyball' OR bobsleigh OR bobsled OR bobsledge OR biathlon OR curling OR 'alpine ski' OR 'ice hockey'/exp OR 'hockey, ice' OR 'ice hockey' OR 'icehockey' OR 'hockey'/exp OR 'field hockey' OR 'hockey' OR luge OR 'speed skating' OR 'ski jumping' OR 'skiing'/exp OR 'cross country skiing' OR 'running, ski' OR 'ski' OR 'ski running' OR 'skiing' OR 'snow skiing' OR 'snowboarding' OR 'dancing'/exp OR 'dancing' OR 'ballet'/exp OR slacklining OR slackline OR Exergames[tiab] OR Virtual Reality[tiab] OR Virtual Reality Exposure Therapy[tiab] OR capoeira OR 'rope skipping' OR 'bicycle'/exp OR 'bicycle' OR 'roller skating'/exp OR 'roller skating' OR 'rollerskating' OR 'skating, roller' OR 'skateboarding'/exp OR 'skateboarding' OR 'skating'/exp OR 'ice skating' OR 'skating' OR 'sport'/mj OR 'competitive gymnastics':ti OR 'competitive sport':ti OR 'sport':ti OR 'sports':ti) AND [embase]/lim NOT ([embase]/lim AND [medline]/lim)

WEB OF SCIENCE

((pediatr$ OR child OR children OR Adolescent OR teen$ OR teenager OR infant) AND ("hearing impairment" OR "impaired hearing" OR "auditory defect" OR "hearing damage" OR "hearing defect" OR "bilateral deafness" OR "deaf" OR "deafness" OR "hearing loss" OR "perception deafness" OR "deafness sensorineural" OR "hearing loss sensorineural" OR "neurosensory deafness" OR "neurosensory hearing impairment" OR "neurosensory hearing loss" OR "sensori-neural deafness" OR "sensori-neural hearing impairment" OR "sensori-neural hearing loss" OR "sensorineural deafness" OR "sensorineural hearing impairment" OR "sensorineural hearing loss" OR "vestibular disorder" OR "disorder vestibular" OR "vestibular disease" OR "vestibular disorder" OR "vestibular disturbance" OR "vestibular dysfunction" OR "vestibular impairment" OR "vestibulopathy") AND ("Track and Field" OR "Racquet Sports" OR "Basket ball" OR Basketball OR boxing OR "aquatic sports" OR "aquatic sport" OR "water sports" OR watersport* OR Bicycling OR cycling OR "fencing (sport)" OR "fencing sport" OR soccer OR "artistic gymnastics" OR "rhythmic gymnastics" OR Golf OR handball OR equestrian OR Hockey OR icehockey OR "Martial Arts" OR "martial art" OR "martial sport" OR "Weight Lifting" OR Wrestling OR Swimming OR "Water Sports" OR "Water Sport" OR pentatl$ OR football OR rugby OR taekwondo OR "taekwon-do" OR "tae kwon do" OR "pistol sport" OR Tennis OR "ping-pong" OR triatlon OR triathlon OR Volleyball OR bobsleigh OR bobsled OR bobsledge OR biathlon OR curling OR "alpine ski" OR "cross-country" OR ski OR luge OR "ice skating" OR "speed skating" OR "Ski jumping" OR skeleton OR Skiing OR Snowboarding OR ballet OR dancing OR Slacklining OR Slackline OR Exergames[tiab] OR Virtual Reality[tiab] OR Virtual Reality Exposure Therapy[tiab] OR capoeira OR "rope skipping" OR bike OR bicycle OR "Roller skating" OR rollerskating OR skateboard OR skateboarding OR skating OR surf))

CENTRAL

(pediatr*[tiab] OR child[mh] OR children[tiab] OR Adolescent[mh] OR Adolescent*[tiab] OR teen*[tiab] OR teenager[tiab] OR child*[tiab] OR infant[tiab]) AND ("hearing impairment"[mh] OR "hearing impairment"[tiab] OR "impaired hearing"[tiab] OR "auditory defect"[tiab] OR "hearing damage"[tiab] OR "hearing defect"[tiab] OR "bilateral deafness"[tiab] OR "deaf"[tiab] OR "deafness"[tiab] OR "hearing loss"[tiab] OR "perception deafness"[tiab] OR "deafness sensorineural"[tiab] OR "hearing loss sensorineural"[tiab] OR "neurosensory deafness"[tiab] OR "neurosensory hearing impairment"[tiab] OR "neurosensory hearing loss"[tiab] OR "sensori-neural deafness"[tiab] OR "sensori-neural hearing impairment"[tiab] OR "sensori-neural hearing loss"[tiab] OR "sensorineural deafness"[tiab] OR "sensorineural hearing impairment"[tiab] OR "sensorineural hearing loss"[tiab] OR "vestibular disorder"[tiab] OR "disorder vestibular"[tiab] OR "vestibular diseases"[mh] OR "vestibular disease"[tiab] OR "vestibular disorder"[tiab] OR "vestibular disturbance"[tiab] OR "vestibular dysfunction"[tiab] OR "vestibular impairment"[tiab] OR "vestibulopathy"[tiab]) AND ("Track and Field"[mh] OR "Track and Field"[tiab] OR "Racquet Sports"[mh] OR "Racquet Sports"[tiab] OR Basketball[mh] OR "Basket

ball"[tiab] OR Basketball[tiab] OR boxing[tiab] OR boxing[mh] OR "aquatic sports"[tiab] OR "aquatic sport"[tiab] OR "water sports"[tiab] OR watersport*[tiab] OR Bicycling[mh] OR Bicycling[tiab] OR cycling[tiab] OR "fencing (sport)"[tiab] OR "fencing sport"[tiab] OR soccer[mh] OR soccer[tiab] OR "artistic gymnastics"[tiab] OR "rhythmic gymnastics"[tiab] OR Golf[mh] OR Golf[tiab] OR handball[tiab] OR equestrian[tiab] OR Hockey[mh] OR Hockey[tiab] OR icehockey[tiab] OR "Martial Arts"[tiab] OR "Martial Arts"[mh] OR "martial art"[tiab] OR "martial sport"[tiab] OR "Weight Lifting"[mh] OR "Weight Lifting"[tiab] OR Wrestling[mh] OR Wrestling[tiab] OR Swimming[mh] OR Swimming[tiab] OR "Water Sports"[mh] OR "Water Sports"[tiab] OR "Water Sport"[tiab] OR pentatl*[tiab] OR football[mh] OR football[tiab] OR rugby[tiab] OR taekwondo[tiab] OR "taekwon-do"[tiab] OR "tae kwon do"[tiab] OR "pistol sport"[tiab] OR Tennis[mh] OR Tennis[tiab] OR "ping-pong"[tiab] OR triatlon[tiab] OR triathlon[tiab] OR Volleyball[mh] OR Volleyball[tiab] OR bobsleigh[tiab] OR bobsled[tiab] OR bobsledge[tiab] OR biathlon[tiab] OR curling[tiab] OR "alpine ski"[tiab] OR "cross-country"[tiab] OR ski[tiab] OR Hockey[tiab] OR luge[tiab] OR "ice skating"[tiab] OR "speed skating"[tiab] OR "Ski jumping"[tiab] OR skeleton[tiab] OR Skiing[mh] OR Skiing[tiab] OR Snowboarding[tiab] OR Dancing[mh] OR ballet[tiab] OR dancing[tiab] OR Slacklining[tiab] OR Slackline[tiab] OR Exergames[tiab] OR Virtual Reality[tiab] OR Virtual Reality Exposure Therapy[tiab] OR capoeira[tiab] OR "rope skipping"[tiab] OR bike[tiab] OR bicycle[tiab] OR "Roller skating"[tiab] OR rollerskating[tiab] OR skateboard[tiab] OR skateboarding[tiab] OR skating[mh] OR skating[tiab] OR surf[tiab] OR sport[majr])

CINAHL

((pediatr* OR "childhood" OR children OR Adolescent* OR "teenager" OR "teen" OR infant*) AND ("hearing impairment" OR "impaired hearing" OR "auditory defect" OR "hearing damage" OR "hearing defect" OR "bilateral deafness" OR "deaf" OR "deafness" OR "hearing loss" OR "perception deafness" OR "deafness sensorineural" OR "hearing loss sensorineural" OR "neurosensory deafness" OR "neurosensory hearing impairment" OR "neurosensory hearing loss" OR "sensori-neural deafness" OR "sensori-neural hearing impairment" OR "sensori-neural hearing loss" OR "sensorineural deafness" OR "sensorineural hearing impairment" OR "sensorineural hearing loss" OR "vestibular disorder" OR "disorder vestibular" OR "vestibular diseases" OR "vestibular disease" OR "vestibular disorder" OR "vestibular disturbance" OR "vestibular dysfunction" OR "vestibular impairment" OR "vestibulopathy")) AND ("Track and Field" OR "Racquet Sports" OR "Basketball" OR "Basket ball" OR "boxing" OR "aquatic sports" OR "water sports" OR watersport* OR "Bicycling" OR "cycling" OR "fencing sport" OR "soccer" OR "artistic gymnastics" OR "rhythmic gymnastics" OR "Golf" OR "handball" OR "equestrian" OR "Hockey" OR "icehockey" OR "Martial Arts" OR "martial art" OR "martial sport" OR "Weight Lifting" OR "Wrestling" OR "Swimming" OR "Water Sports" OR "Water Sport" OR pentatl* OR "football" OR "rugby" OR "taekwondo" OR "taekwon-do" OR "tae kwon do" OR "pistol sport" OR "Tennis" OR "ping-pong" OR "triathlon" OR "triathlon" OR "Volleyball" OR "bobsleigh" OR "bobsled" OR "bobsledge" OR "biathlon" OR "curling" OR "alpine ski" OR "cross-country" OR "ski" OR "Hockey" OR "luge" OR "ice skating" OR "speed skating" OR "Ski jumping" OR "skeleton" OR "Skiing" OR "Snowboarding" OR "ballet" OR "dancing" OR "Slacklining" OR "Slackline" OR "Exergames" OR "Virtual Reality" OR "Virtual Reality Exposure Therapy" OR "capoeira" OR "rope skipping" OR "bike" OR "bicycle" OR "Roller skating" OR "rollerskating" OR "skateboard" OR "skateboarding" OR "skating" OR "surf")

SCOPUS

(TITLE-ABS (pediatr* OR "childhood" OR children OR adolescent* OR "teenager" OR "teen" OR infant*) AND TITLE-ABS ("hearing impairment" OR "impaired hearing" OR "auditory defect" OR "hearing damage" OR "hearing defect" OR "bilateral deafness" OR "deaf" OR "deafness" OR "hearing loss" OR "perception deafness" OR "deafness sensorineural" OR "hearing loss sensorineural" OR "neurosensory deafness" OR "neurosensory hearing impairment" OR "neurosensory hearing loss" OR "sensori-neural deafness" OR "sensori-neural hearing impairment" OR "sensori-neural hearing loss" OR "sensorineural deafness" OR "sensorineural hearing impairment" OR "sensorineural hearing loss" OR "vestibular disorder" OR "disorder vestibular" OR "vestibular diseases" OR "vestibular disease" OR "vestibular disorder" OR "vestibular disturbance" OR "vestibular dysfunction" OR "vestibular impairment" OR "vestibulopathy")) AND TITLE-ABS ("Track and Field" OR "Racquet Sports" OR "Basketball" OR "Basket ball" OR "boxing" OR "aquatic sports" OR "water sports" OR watersport* OR "Bicycling" OR "cycling" OR "fencing sport" OR "soccer" OR "artistic gymnastics" OR "rhythmic gymnastics" OR "Golf" OR "handball" OR "equestrian" OR "Hockey" OR "icehockey" OR "Martial Arts" OR "martial art" OR

"martial sport" OR "Weight Lifting" OR "Wrestling" OR "Swimming" OR "Water Sports" OR "Water Sport" OR pentatl* OR "football" OR "rugby" OR "taekwondo" OR "taekwon-do" OR "tae kwon do" OR "pistol sport" OR "Tennis" OR "ping-pong" OR "triathlon" OR "triathlon" OR "Volleyball" OR "bobsleigh" OR "bobsled" OR "bobsledge" OR "biathlon" OR "curling" OR "alpine ski" OR "cross-country" OR "ski" OR "Hockey" OR "luge" OR "ice skating" OR "speed skating" OR "Ski jumping" OR "skeleton" OR "Skiing" OR "Snowboarding" OR "ballet" OR "dancing" OR "Slacklining" OR "Slackline" OR "Exergames" OR "Virtual Reality" OR "Virtual Reality Exposure Therapy" OR "capoeira" OR "rope skipping" OR "bike" OR "bicycle" OR "Roller skating" OR "rollerskating" OR "skateboard" OR "skateboarding" OR "skating" OR "surf")

LILACS

(tw:pediatr$ OR tw:"childhood" OR tw:children OR tw:Adolescent$ OR tw:"teenager" OR tw:"teen" OR tw:infant$ or tw:crianca$ or tw:nino$ OR mh:adolescent OR mh:child) AND (mh:"Hearing Loss" OR tw:"hearing impairment" OR tw:"Pérdida Auditiva" OR tw:"Perda Auditiva" OR tw:"impaired hearing" OR tw:"auditory defect" OR tw:"hearing damage" OR tw:"hearing defect" OR tw:"bilateral deafness" OR tw:"deaf" OR tw:"deafness" OR tw:"hearing loss" OR tw:"perception deafness" OR tw:"deafness sensorineural" OR tw:"hearing loss sensorineural" OR tw:"neurosensory deafness" OR tw:"neurosensory hearing impairment" OR tw:"neurosensory hearing loss" OR tw:"sensori-neural deafness" OR tw:"sensori-neural hearing impairment" OR tw:"sensori-neural hearing loss" OR tw:"sensorineural deafness" OR tw:"sensorineural hearing impairment" OR tw:"sensorineural hearing loss" OR tw:"vestibular disorder" OR tw:"disorder vestibular" OR tw:"vestibular diseases" OR tw:"vestibular disease" OR tw:"vestibular disorder" OR tw:"vestibular disturbance" OR tw:"vestibular dysfunction" OR tw:"vestibular impairment" OR tw:"vestibulopathy" OR tw:"Doenças Vestibulares" OR tw:"Enfermedades Vestibulares" OR mh:"Vestibular Diseases") AND (mh:"Track and Field" OR tw:"Track and Field" OR tw:atletismo OR tw:"Esportes com Raquete" OR tw:"Deportes de Raqueta" OR mh:"Racquet Sports" OR tw:"Racquet Sports" OR tw:Basketball OR tw:Baloncesto OR tw:Basquetebol OR tw:basquete OR mh:"Basketball" OR tw:"Basket ball" OR mh:boxing OR tw:boxeo OR tw:boxe OR tw:"boxing" OR mh:"Water Sports" OR tw:"Deportes Acuáticos" OR tw:"Esportes Aquáticos" OR "water sports" OR watersport$ OR mh:Bicycling OR tw:ciclismo tw:Bicycling OR tw:cycling OR tw:"fencing sport" OR tw:esgrima OR tw:soccer OR tw:futebol OR tw:Fútbol OR tw:football OR tw:"artistic gymnastics" OR tw:"ginástica artística" OR tw:"gimnasia artística" OR tw:"Gimnasia Rítmica" OR tw:"ginástica rítmica" OR tw:"rhythmic gymnastics" OR mh:Golf OR tw:Golf OR tw:Golfe OR tw:handball OR tw:handebol OR tw:balonmano OR tw:equestrian OR tw:ecuestre OR tw:hipismo OR mh:Hockey OR tw:Hockey OR tw:Hoquei OR tw:icehockey OR mh:"Martial Arts" OR TW:"Artes Marciales" OR TW:"Artes Marciais" OR tw:"Martial Arts" OR tw:"martial art" OR tw:"martial sport" OR mh:"Weight Lifting" OR tw:"Levantamento de Peso" OR tw:"Levantamiento de Peso" OR mh:Wrestling OR tw:"Lucha Olímpica" OR tw:"Luta Olímpica" OR tw:wrestling OR tw:Swimming OR mh:Swimming OR TW:Natación OR tw:Natação OR tw:pentatl$ OR TW:rugby OR tw:"Fútbol Americano" OR tw:"Futebol Americano" OR mh:Football OR TW:taekwondo OR tw:"taekwon-do" OR tw:"tae kwon do" OR tw:"pistol sport" OR tw:"tiro esportivo" OR mh:Tennis OR tw:tennis OR tw:tenis OR tw:"ping-pong" OR tw:triathlon OR tw:triatlon OR mh:Volleyball OR tw:Volleyball OR tw:voleibol OR tw:volei OR tw:bobsleigh OR tw:bobsled OR tw:bobsledge OR tw:biathlon OR tw:biatlo OR tw:curling OR tw:"alpine ski" OR tw:"cross-country" OR tw:ski OR tw:"luge" OR tw:"ice skating" OR tw:"speed skating" OR tw:"Ski jumping" OR tw:"skeleton" OR mh:Skiing OR tw:esqui OR tw:skiing OR tw:Snowboarding OR “tw:dancing OR mh:dancing OR tw:ballet OR tw:danca OR tw:Slacklining OR tw:Slackline OR tw:"exergames" OR tw: "virtual reality" OR tw: "virtual reality exposure therapy" OR tw:capoeira OR tw:"rope skipping" OR tw:"pular corda" OR tw:bike OR tw:bicycle OR mh:ciclismo OR tw:ciclismo OR tw:"Roller skating" OR tw:rollerskating OR tw:skateboard OR tw:skateboarding OR tw:skating OR tw:surf)

PEDro

((pediatr* OR "childhood" OR children OR Adolescent* OR "teenager" OR "teen" OR infant*) AND ("hearing impairment" OR "impaired hearing" OR "auditory defect" OR "hearing damage" OR "hearing defect" OR "bilateral deafness" OR "deaf" OR "deafness" OR "hearing loss" OR "perception deafness" OR "deafness sensorineural" OR "hearing loss sensorineural" OR "neurosensory deafness" OR "neurosensory hearing impairment" OR "neurosensory hearing loss" OR "sensori-neural deafness" OR "sensori-neural hearing impairment" OR "sensori-neural hearing loss" OR "sensorineural deafness" OR "sensorineural hearing impairment" OR "sensorineural hearing loss" OR "vestibular disorder" OR "disorder vestibular" OR "vestibular diseases" OR "vestibular disease" OR "vestibular disorder" OR "vestibular disturbance" OR "vestibular dysfunction" OR "vestibular impairment" OR "vestibulopathy")) AND ("Track and Field" OR "Racquet Sports" OR "Basketball" OR "Basket ball" OR "boxing" OR "aquatic sports" OR "water sports" OR watersport* OR "Bicycling" OR "cycling" OR "fencing sport" OR "soccer" OR "artistic gymnastics" OR "rhythmic gymnastics" OR "Golf" OR "handball" OR "equestrian" OR "Hockey" OR "icehockey" OR "Martial Arts" OR "martial art" OR "martial sport" OR "Weight Lifting" OR "Wrestling" OR "Swimming" OR "Water Sports" OR "Water Sport" OR pentatl* OR "football" OR "rugby" OR "taekwondo" OR "taekwon-do" OR "tae kwon do" OR "pistol sport" OR "Tennis" OR "ping-pong" OR "triathlon" OR "triathlon" OR "Volleyball" OR "bobsleigh" OR "bobsled" OR "bobsledge" OR "biathlon" OR "curling" OR "alpine ski" OR "cross-country" OR "ski" OR "Hockey" OR "luge" OR "ice skating" OR "speed skating" OR "Ski jumping" OR "skeleton" OR "Skiing" OR "Snowboarding" OR "ballet" OR "dancing" OR "Slacklining" OR "Slackline" OR "exergames" OR "virtual reality" OR "virtual reality exposure therapy" OR "capoeira" OR "rope skipping" OR "bike" OR "bicycle" OR "Roller skating" OR "rollerskating" OR "skateboard" OR "skateboarding" OR "skating" OR "surf")

Google Scholar

(("sports" OR "motor activity" OR "exergames" OR "virtual reality" OR "virtual reality exposure therapy") AND ("child" OR "adolescent") AND ("vestibular diseases" OR "hearing impairment"))
